# 2G12/PGT-binding yeast glycoprotein Gp38 elicits mannose-specific HIV-1 Env cross-reactive antibodies

**DOI:** 10.1186/1742-4690-9-S2-P357

**Published:** 2012-09-13

**Authors:** H Zhang, H Fu, B Liu, RJ Luallen, RW Doms, DF Smith, Y Geng

**Affiliations:** 1 ProSci Inc., Poway, CA, USA; 2 University of Pennsylvania, Philadelphia, PA, USA; 3 Emory University , Atlanta, GA, USA

## Background

The increasing numbers of broad neutralizing antibodies (bNAbs) that target carbohydrates of HIV envelops highlight the importance of designing immunogens to elicit such types of bNAbs for an effective HIV vaccine.

## Methods

PGT bNAbs-cross reactive proteins were detected by Western blots and identified by nano-LC-MS/MS. Rabbit antisera were raised with single PGT bNAbs-reactive yeast glycoprotein, and tested by ELISA, Western blots and glycan microarray. HIV-1 pseudoviruses were generated in 293T cells, and neutralization assay was performed using TZM-bl cells.

## Results

Using the newly identified, glycan-specific PGT bNAbs to search for their binders from a triple mutant(TM) strain of Saccharomyces cerevisiae, we found that the PGT bNAbs not only bind to the previously identified 2G12-reactive glycoproteins but also recognize several unknown proteins in TM yeast. One of them was identified as a short version of Gp38 with N-terminus truncation. Based on immunization of rabbits with various formulations and strategies, we found that a high titer of HIV-1 Env cross-reactive antibodies was induced when using a promiscuous T-cell epitope peptide conjugated Gp38 in a formulation with a Toll-like receptor 2 agonist and aluminum salts. The Gp38-elicited antibodies could bind to a broad range of monomeric gp120s from HIV and SIV. Moreover, the antibodies could also efficiently neutralize HIV-1 pseudoviruses when the viruses were produced in the presence of a mannosidase inhibitor kifunensine, which enriches high-mannose Man9GlcNAc2 N-linked glycans. Glycan microarray analysis showed that these antibodies bind to the synthetic Manα1,2-Manα1,2-Man containing oligosaccharides.

## Conclusion

These data suggest that yeast glycoprotein Gp38, as well as its truncated form, is an efficient binder to the glycan-specific HIV bNAbs, and that Gp38 is able to induce a strong glycan-specific HIV-reactive antibody response when incorporated with appropriate adjuvants. These results encourage us to further explore the strategies to induce 2G12/PGT-like antibodies using Gp38 as well as its truncated form.

